# Enhancing the Neuroprotection Potential of Edaravone in Transient Global Ischemia Treatment with Glutathione- (GSH-) Conjugated Poly(methacrylic acid) Nanogel as a Promising Carrier for Targeted Brain Drug Delivery

**DOI:** 10.1155/2023/7643280

**Published:** 2023-02-21

**Authors:** Faezeh Mozafari, Hamid Rashidzadeh, Soroush Bijani, Faezeh Zare-Molaei, Ziba Islambulchilar, Hossein Danafar, Ali Kalantari-Hesari, Ali Ramazani, Mir-Jamal Hosseini

**Affiliations:** ^1^Zanjan Applied Pharmacology Research Center, Zanjan University of Medical Sciences, Zanjan, Iran; ^2^Department of Pharmacology and Toxicology, School of Pharmacy, Zanjan University of Medical Sciences, Zanjan, Iran; ^3^Pharmaceutical Biotechnology Research Center, School of Pharmacy, Zanjan University of Medical Sciences, Zanjan, Iran; ^4^Pharmaceutical Biomaterials Department, School of Pharmacy, Zanjan University of Medical Sciences, Zanjan, Iran; ^5^Department of Pharmaceutics, Faculty of Pharmacy, Tabriz University of Medical Sciences, Tabriz, Iran; ^6^Department of Pathobiology, Faculty of Veterinary Sciences, Bu-Ali Sina University, Hamedan, Iran

## Abstract

Ischemic stroke is the most common among various stroke types and the second leading cause of death, worldwide. Edaravone (EDV) is one of the cardinal antioxidants that is capable of scavenging reactive oxygen species, especially hydroxyl molecules, and has been already used for ischemic stroke treatment. However, poor water solubility, low stability, and bioavailability in aqueous media are major EDV drawbacks. Thus, to overcome the aforementioned drawbacks, nanogel was exploited as a drug carrier of EDV. Furthermore, decorating the nanogel surface with glutathione as targeting ligands would potentiate the therapeutic efficacy. Nanovehicle characterization was assessed with various analytical techniques. Size (199 nm, hydrodynamic diameter) and zeta potential (-25 mV) of optimum formulation were assessed. The outcome demonstrated a diameter of around 100 nm, sphere shape, and homogenous morphology. Encapsulation efficiency and drug loading were determined to be 99.9% and 37.5%, respectively. *In vitro* drug release profile depicted a sustained release process. EDV and glutathione presence in one vehicle simultaneously made the possibility of antioxidant effects on the brain in specific doses, which resulted in elevated spatial memory and learning along with cognitive function in Wistar rats. In addition, significantly lower MDA and PCO and higher levels of neural GSH and antioxidant levels were observed, while histopathological improvement was approved. The developed nanogel can be a suited vehicle for drug delivery of EDV to the brain and improve ischemia-induced oxidative stress cell damage.

## 1. Introduction

Though acute ischemic stroke has moved from the second cause of mortality to fourth one in the United States, the long-term effects of this disorder are still the second leading cause of death among individuals [1]. The main factors, which affect the chance of an ischemic event, are gender, age, hypertension, hyperlipidemia, diabetes, smoking, and ventricular fibrillation [2]. Though numerous medications were investigated to minimize poststroke complications, none succeeded to present complete and reliable prevention of neural damage and improve the quality of life of the patients.

Ischemic stroke deprives the brain from its sufficient source of blood flow, which would lead to the alteration of several biological factors in poststroke state, namely, overproduction of free radicals, shortage of oxygen supply, excitotoxicity, overaccumulation of intracellular calcium and sodium, activation of inflammatory pathways, and mitochondrial dysfunction [3]. The formation of reactive oxygen species (ROS) is the first step in mitochondrial dysfunction, which could lead to the activation of endothelial cells and glial cells having a direct impact on the overproduction of proinflammatory cytokines and chemokines [4].

The aforementioned pathway does not provide us with many medications, to treat ischemic stroke. The majority of available medications have mainly focused on thrombolytic pathways, providing some promising effects. Besides the narrow time window, thrombolytics are associated with life-threatening side effects, including the deformity of the blood-brain barrier and the increasing risk of hemorrhagic bleeding, leading to more neural damage [5]. The application of free radical scavengers and excitatory amino acids is another medical approach in this field [6].

Free radical overaccumulation in poststroke state is highly associated with protein carbonization, DNA damage, and peroxidation of lipids leading to neural cell necrosis and homeostatic impairment of neural tissue, mainly the blood-brain barrier and vessels [7]. Counterneutralization of reactive species inside the cell is accomplished by consumption of biological antioxidants such as glutathione. Oxidative stress is responsible for various CNS diseases, and its role in neurodegeneration propagation and initiation is well established. In case of desirable bioavailability, small antioxidant molecules are highly beneficial for alleviation of CNS oxidative stress by neutralization of oxidative stressors [8]. Targeting the root cause of neurodegeneration by antioxidants is a promising therapeutic approach for ischemic stroke.

Edaravone is a strong antioxidant, which has proven its neuroprotective effects in ALS through neutralizing both water-soluble and insoluble peroxyl radicals and thus slowing the pattern of movement loss by 33% [9, 10]. EDV has demonstrated a probable improving effect on the outcome of ischemic stroke management when applied with a thrombolytic agent [11], and in a clinical study, EDV indicated a trend of effectiveness with a moderately reliable outcome in acute stroke [12]. While managing to suppress the accumulation and surge of ROS, EDV modulated the overexpression of various inflammatory-related factors, including COX-2, NF-*κ*B, and interleukins [13]. In this study, EDV was utilized as a free radical scavenger agent to observe the probable ameliorating effects on poststroke symptoms. However, EDV is far from being perfect for successful application in treatment of ischemic stroke due to its poor water solubility, low stability, and bioavailability in aqueous media. One promising strategy to overcome EDV-associated drawbacks is implementation of nanotechnology-based drug carrier. Among various types of drug carrier, polymetacrylic acid (PMAA) nanogel owing to its unique properties such as high stability in aqueous media, high biocompatibility, ease of functionalization by bioconjugation of active targeting agents, high loading capacity, and controlled release of its payload has gained remarkable attention in delivery of numerous bioactive agents [14]. Moreover, to avoid multiple injections and increase the site-specific delivery of EDV to the brain tissue, glutathione as targeting ligands was decorated onto the surface of PMAA nanogel. It is well documented that targeted drug delivery could improve the therapeutic potential as well as the efficacy of the treatment [14]. In this regard, glutathione-conjugated PMAA was developed as a drug vehicle to deliver EDV (GSH-PMAA-EDV), to investigate its probable ameliorating effect on various factors relating to the behavioral and biochemical performances of ischemic stroke rodent models. Novel object recognition (NOR) test and spontaneous alternation T-maze, as an animal-friendly behavioral test, were precisely and accurately conducted to assess the reproducibility of rat memory performance and working memory, respectively.

## 2. Materials and Methods

### 2.1. Materials

Methacrylic acid (MAA) monomer and bisacrylamide (Bis AM) as a cross-linker were obtained from Sigma-Aldrich, and they were purified prior to use through distillation and recrystallization process. N-Hydroxysuccinimide (NHS), N-(3-dimethylaminopropyl)-N′-ethylcarbodiimide hydrochloride (EDC), and azobisisobutyronitrile (AIBN) were purchased from Sigma-Aldrich. EDV and glutathione were also purchased from Merck. All other utilized chemicals, reagents, and solvents were of analytical grade or good quality lab chemical grade.

### 2.2. Synthesis of Poly(methacrylic acid) (PMAA) Nanogel

PMAA nanogel was prepared according to our previous research by using the reflux-precipitation polymerization method [14]. In brief, 0.042 mmol of AIBN, 0.172 mmol of Bis AM, and 3.485 mmol of MAA were introduced in a dried 50 mL single-necked flask containing 20 ml of acetonitrile. Then, 15 min of ultrasonic agitation was applied to dissolve the mixture in acetonitrile. The obtained homogeneous reaction mixture was then placed in the oil bath and heated to reach the boiling point of the acetonitrile for 1 h. The crude products were obtained using ultracentrifugation (15,000 rpm for 10 min) and dried overnight under a vacuum at 50°C.

### 2.3. Conjugation of Glutathione onto the PMAA (GSH-PMAA)

One promising strategy in coupling the amine-containing compounds to the carboxylic acids is a carbodiimide coupling method. Glutathione and PMAA nanogel have amine and carboxylic acid groups, respectively. The conjugation reaction proceeded through an amide formation, and it was carried out according to our previous research with some modifications [14]. In this case, N-ethyl-N′-(3-dimethylaminopropyl) carbodiimide hydrochloride/N-hydroxysuccinimide (EDC.HCl/NHS), activated carboxylic acid groups of PMAA nanogel (PMAA-COOH), and then the amine group of GSH (GSH-NH_2_) react with the activated carboxylic acids. Typically, ultrasonic agitation was applied to disperse 30 mg PMAA nanogel in 20 ml of acetonitrile. After that, in order to activate the carboxylic acids of PMAA, 0.25 mmol of EDC.HCl/NHS was added to the above dispersion and stirred for 3-6 h, at room temperature. Finally, 0.03 mmol of GSH-NH_2_ was added to the activated PMAA-COOH dispersion and continued stirring for 24 h, at room temperature. The prepared GSH-PMAA nanogel was obtained using ultracentrifugation (18,000 rpm for 15 min) and freeze-dried.

### 2.4. Preparation of GSH-PMAA-EDV

EDV is a potent hydrophobic drug with unique neuroprotective activity and capability of scavenging the free radicals in the treatment of ischemic stroke. In order to enhance the uptake of EDV in the brain and improve its solubility, GSH-PMAA-EDV was prepared. Loading capacity was also carried out based on a previously published study [14]. In brief, a known amount of dried GSH-PMAA (10 mg) was dispersed in distilled water with the aid of ultrasonic agitation; after that, 6 mg of EDV was added into the homogeneous dispersion of GSH-PMAA nanogel and continued stirring for 24 h, at room temperature. Then, the GSH-PMAA-EDV was obtained using ultracentrifugation, freeze-dried, and kept at -4-8°C. Finally, the amount of EDV encapsulated within GSH-PMAA was calculated by a UV-visible spectrophotometer (UV-visible spectrophotometer T 80+) at 244 nm.

### 2.5. *In Vitro* Drug Release Study

One of the most frequently used techniques in the determination of *in vitro* drug release behavior of any developed drug delivery system is a dialysis bag technique. Briefly, in a dialysis bag, GSH-PMAA-EDV (1 mg) was dispersed in phosphate buffer solution (2 ml/pH 7.4), and then, it was immersed in 25 ml of phosphate buffer solution to be dialyzed while gently shaken at 250 rpm at 37°C. All these steps were employed for bare drug (EDV). Once a dialysis bag containing GSH-PMAA-EDV or EDV was immersed into the PBS solution, the drug release was assumed to start. At a predetermined time interval, a known amount of the surrounding media (2 ml) was taken from the reservoir and replaced with 2 ml of fresh buffer medium to keep the reservoir volume constant, and the amounts of EDV released from the GSH-PMAA nanogel were analyzed using UV-vis at 244 nm (UV-visible spectrophotometer T 80+).

### 2.6. Characterization

Various techniques were used to fully characterize for developed GSH-PMAA-EDV structure. For instance, in terms of morphological characterization and size distribution, transmission electron microscopy (TEM, FEI 120 kV) along with atomic force microscopy (AFM) was applied. Moreover, to characterize the structure of GSH-PMAA nanogel as well as confirm the GSH molecule conjugation onto PMAA, Fourier transform infrared spectroscopy (FTIR) technique (Bruker, Tensor 27, USA) and proton nuclear magnetic resonance (^1^H NMR) (BRUKER DRX-250 AVANCE) were used.

### 2.7. Size and Zeta Potential

Both size and zeta potential of GSH-PMAA-EDV nanogel were determined using dynamic light scattering (DLS) on a nano/zeta sizer (Malvern Instruments, Worcestershire, UK, ZEN 3600 model Nano ZS).

### 2.8. Animals

Wistar male rats weighted 200-220 g were purchased from the Pasteur Institute, Tehran, Iran. They were housed under laboratory conditions, meaning 12 hours of light/dark cycle, free access to food/water, and 21-23°C temperature. All tests were performed during daylight from 10 AM to 2 PM according to NIH guidelines and under the approval of the animal ethic committee of Zanjan University of Medical Sciences (ethical code: ZUMS.REC.1398.0132).

### 2.9. Experimental Design

**Figure 1:**
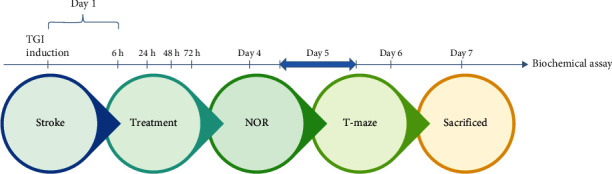
Timeline of procedure, treatment, and evaluation of behavioral and biochemical analyses.

Animals were randomly divided into 8 groups with each group consisting of 10 rats (80 in total): (1) the control group only received normal saline; (2) normal or nonstroke rats, which received only GSH-PMAA-EDV at the highest utilized dose (250 *μ*g/kg); (3) ischemic stroke was induced by transient global ischemia (TGI) model; (4) ischemic stroke rats, which received only GSH-PMAA-EDV at a highest utilized dose (250 *μ*g/kg); and (5-8) ischemia-induced rats which received GSH-PMAA-EDV with different equivalent doses of EDV (5, 20, 40, and 250 *μ*g/kg) (Figure 1).

### 2.10. Ischemic Stroke Induction Procedure

For induction of ischemic stroke, male Wistar rats went under transient global ischemia (TGI), a method widely utilized to replicate the surge of oxidative stress, neural damage, and immunoinflammatory aspects of stroke in the central nervous system [15, 16]. The method consists of clamping common carotid arteries for 20 minutes after gently opening through neck soft tissues and stitching the surgical site at the end. The first treatment was started 6 h after TGI induction technique, followed by intraperitoneal injection at 24, 48, and 72 h after surgery. Twenty-four hours after the last treatment, animals underwent behavioral tests (novel object recognition and T-maze tests), and 7 days after TGI induction, animals were sacrificed for tissue extraction.

### 2.11. Behavioral Test

#### 2.11.1. Novel Object Recognition (NOR) Test

This test is designed to demonstrate the tendency of rodents to explore unfamiliar objects, in the context of a square-shaped transparent box made of Plexiglas (70 × 70 × 30 cm) in their light phase (10:00 AM-2:00 PM). Rats were not habituated to open field apparatus, and as the first step, they were placed in the testing room for 30 minutes. Additionally, all animals were handled by experimenter for 7 days before familiarization and for the duration of 30 seconds each to acclimate animal to the housing room. Spatial cues with contrasting colors were placed on the walls in a room with dim light at 15 lux in center, adjusted with panel control. Three different objects in triplicate were chosen for the familiarization phase, and two of them were randomly chosen for each rat to be held on the box floor by a double-sided tape, 5 cm away from the walls. Objects were different in shape, color, and texture and fixed to avoid rats from moving objects: falcon, yellow, and green cubic plastic (with 5 cm height and 15 cm width) laboratory bottle. Between Lego bricks, blue cubic plastics, and hamburger tower, an object with a maximized difference was chosen for the test session, considering the prior object used for the first session.

In the first phase, animals were placed in the box to observe identical objects for 10 minutes. In the test session, after 24 hours of intersession intervals, an unfamiliar one replaced one object, and the animal was allowed to explore the field freely for another 10 minutes. Animals were placed into an open field with head opposite to objects and returned to the cage after each session. Sniffing, touching, or looking at objects less than 2 cm distance from it was considered as an explorative behavior which was assessed by the experimenter, as demonstrated in recorded videos. Boxes were carefully disinfected with 70% alcohol and dried between sessions to eliminate olfactory traces and avoid bias in the learning process. Time spent for total object exploring was held in familiarity phase to assure that the stress level was low enough for rodent to learn; then, the discrimination ratio was calculated by dividing the time rodent spent exploring the new object to the total time the rodent explored both objects in test phase [17]. All behavioral tests were executed adhering to nature protocol to improve reliability of results [18]. The box was placed on dark paper to minimize stressors, and videos were recorded in the absence of the experimenter for further analysis.

#### 2.11.2. T-Maze Spontaneous Alternation

Before the experiment started, rats were habituated to the experimenter and then left in the testing room with dim light for 10 minutes for preparation before the experiment. T-maze was constructed manually from medium-density fiberboard (MDF) with dimensions mentioned in nature protocol (except that wall height was 50 cm) for rats, walls painted in black with slides designed in the outer layer of the wall [17]. Five days after surgery was conducted, animals were placed at the bottom part of a T-shaped maze with two arms open to choose and enter into sample phase. Then, they were trapped for 30 seconds in goal arm and were gently removed afterward and put in their home cage. The entrance of the rat to the goal arm was considered when the animal was in the arm with all paws to the tip of the tail. During retention and intertrial intervals, maze walls were cleaned with 70% ethanol with paper, and bedding was removed and replaced with a thin layer of woodchip to remove the influence of odor on alternation. For the choice phase, after 2 minutes of retention interval, the rat was placed on the same former spot, facing away from goal arms, without any closed door, and free to choose each arm. Three trials per rat were performed for 2 consecutive days, in an enclosed-arm maze with discrete trials to assess working memory and hippocampus dysfunction/lesion. All trials were completed in 3 minutes, each. The alternation factor was calculated by dividing the number of alternation by the total number of trials performed [12]. Side preference was calculated by the frequency of choosing one less favorable arm divided by the number of phases (in this study 12 phases).

### 2.12. Tissue Collection

Rats were anesthetized, their ribs were cut, and tissue reperfusion was conducted with normal saline intracardiac infusion (left ventricle) until blood was totally cleared, flowing out of the right atrium. After the hippocampus was dissected, they were frozen on dry ice and then stored at -80°C for further biochemical analysis. Extracted brain tissues were kept in 10% formalin for histological analysis [19].

### 2.13. Biochemical Assays

#### 2.13.1. Evaluation of Lipid Peroxidation (LPO)

The accumulation of ROS leads to the transformation of polyunsaturated fatty acids to aldehydes mainly malonaldehyde (MDA). To measure the production of LPO, the amount of MDA was measured by reaction with thiobarbituric acid (TBARs) with absorption at 532 nm [20].

#### 2.13.2. Ferric Reducing Antioxidant Power (FRAP)

The FRAP test is designed to measure the total level of cellular antioxidants using 2,4,6-Tri(2-pyridyl)-s-triazine (TPTZ) as the sensitive reagent. The rise in absorbance at 593 nm was determined by the antioxidant activity of the generated ferrous ions [21].

#### 2.13.3. Redacted Glutathione (GSH) Assay

GSH acts as the main endogenous antioxidant in neural tissue and is the first marker to alter under pathophysiological oxidative stress. The complex formed by reacting GSH with 5,5′-dithiobis(2-nitrobenzoic acid) had the highest absorption at 421 nm [22].

#### 2.13.4. Protein Carbonyl (PCO)

PCO counts as another byproduct of oxidative stress, increasing pattern. This measurement was based on the PCO reaction with 2,4-dinitrophenylhydrazine, and the absorbance was measured at 365 nm [23].

### 2.14. Histopathological Analysis

At the end of the prescribed period, animals were euthanized by using ketamine-xylazine, and brain samples were taken. Brains were fixed (in buffered formalin 4%) for histopathological studies using dehydration (respectively, with alcohols of 50, 70, 80, and 90% and absolute alcohol), clearing (using xylene), paraffin embedding, blocking, sectioning to 5–7 *μ*m thickness (using Leica RM2025 Microtome), mounting on slides, and hematoxylin and eosin staining (H&E) processes. Histopathological studies included the neuron perikaryon, nerve fibers and myelin sheaths, matrix of the brain, CNS (central nervous system), glial cells (astrocytes, oligodendrocytes, and microglia), and abnormal features. Imaging was carried out with a digital camera Dino-Lite lens and Dino-Capture 2 software [24].

### 2.15. Statistical Analysis

Results were expressed as mean ± SD, and R studio programming software was used for statistical analyses. Comparison between the groups was performed using one-way analysis of variance (ANOVA) followed by Bonferroni's multiple comparisons, and a *P* value lesser than 0.05 was considered statistically significant. Plots were generated using the Matplotlib Library in Python.

## 3. Results

### 3.1. Verification of PMAA and GSH Molecule Synthesis Using FTIR Output

**Figure 2:**
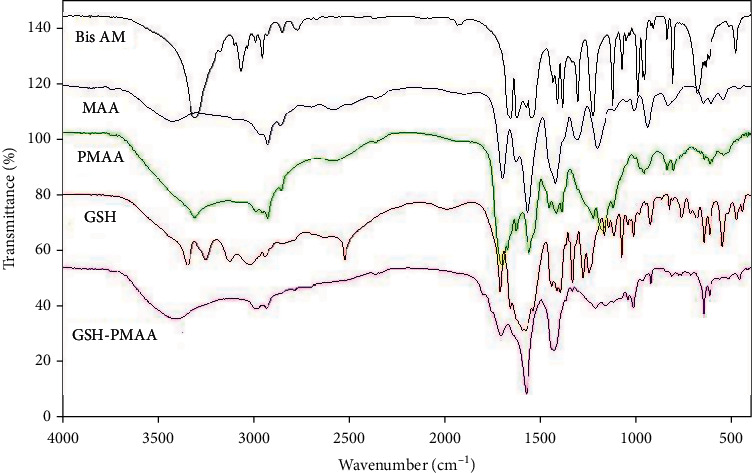
FTIR spectra of Bis AM, MAA, PMAA, GSH, and GSH-PMAA nanoparticles.

The FTIR spectrum of Bis AM is given in Figure 2; the absorption band at 3308 cm^−1^ is related to the stretching vibrations of the amine group (N-H) in the Bis AM molecule. Also, the absorption related to the stretching vibrations of the vinyl (C=CH) and aliphatic (-C-H) group in this compound was observed at 3067 cm^−1^ and 2956 cm^−1^, respectively. The carbonyl group of this compound also shows an absorption band at 1657 cm^−1^ [25]. MAA is one of the other main components in the preparation of gel and nanogel, and as a monomer, it forms the major bulk of the mass of nanogel. The spectrum of this substance is given in Figure 2 as well. The absorption band at ~1700 cm^−1^ is related to the stretching vibration of the carboxylic acid group; also, this acid group shows a broad absorption band at 2500-3500 cm^−1^. The absorption band at 1660 cm^−1^ is related to the stretching vibration of the vinyl group (C=C). FTIR spectrum of PMAA nanogel after polymerization is given in Figure 2. As depicted in this figure, the vinyl band related to Bis AM and MAA has been disappeared, which indicates that the polymerization reaction successfully proceed, while the absorption bands related to the carbonyl group present in Bis AM and MAA are seen with a slight shift in 1668 cm^−1^ and 1705 cm^−1^, respectively.

The FTIR spectrum of this molecule is also given in Figure 2. The absorption bands at 3200-3500 cm^−1^ are related to the amine groups present in the GSH compound. Also, the absorption bands at 1587 cm^−1^ and 1713 cm^−1^ are related to carbonyl groups, and the absorption band at about 2500 cm^−1^ is related to the (-SH) group [26]. The spectrum of the final nanogel after binding GSH as a targeted ligand on the surface of the PMAA nanogel is given in Figure 2. The strong absorption band at ~1678 cm^−1^ indicates the formation of an amide bond between the amine group of GSH and carboxylic acids on the surface of the PMAA nanogel, so it can be concluded that GSH has been successfully conjugated on the nanogel [27].

### 3.2. Verification of PMAA and GSH Molecule Synthesis Using HNMR Output

**Figure 3:**
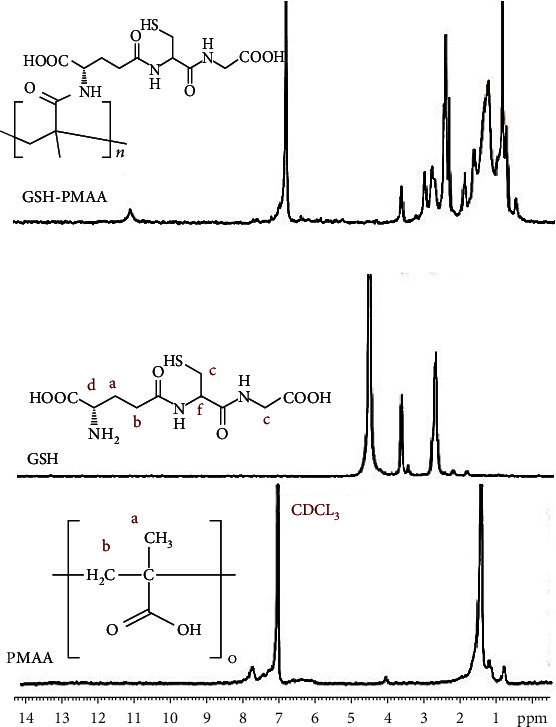
HNMR spectra of PMAA, GSH, and GSH-PMAA.

Another powerful technique in the characterization of GSH-PMAA nanogel is ^1^H NMR. ^1^H NMR spectra related to PMAA nanogel, GSH, and PMAA conjugated with GSH are shown in Figure 3. The peaks in 1-2 ppm are related to saturated hydrogens of methyl (-CH_3_) and methylene (-CH_2_) groups present in PMAA and GSH-PMAA spectra. Also, the absence of a peak at 5-7 ppm, which is characteristic of the vinyl group, confirms that PMAA has been successfully synthesized. Since the spectrum of this compound was taken in CDCL_3_ solvent, the peak related to the solvent is also observed at 7.25 ppm (Figure 3). The characteristic peaks of GSH are given in Figure 2. Also, all the protons of GSH, which are marked from (a) to (f) in Figure 3, appeared at about 2.00 ppm, 2.35 ppm, 2.80 ppm, 3.60 ppm, 3.70 ppm, and 4.60 ppm, respectively.

### 3.3. The Verification of Size, Shape, and Dispersity of GSH-PMAA-EDV Nanogel

**Figure 4:**
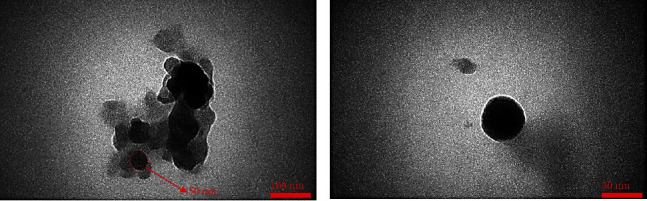
Particle size and morphology of GSH-PMAA using TEM at different magnification scale bars.

Morphology and particle size of developed GSH-PMAA nanogel were investigated using TEM and AFM. GSH-PMAA nanogel was monodispersed and spherical in shape with a mean diameter smaller than 50 nm as depicted in Figure 4.

**Figure 5:**
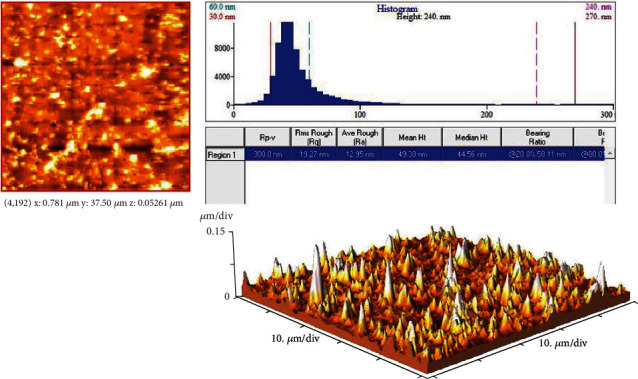
AFM image and morphology of GSH-PMAA.

AFM images also clearly demonstrated that developed nanoparticles were spherical in shape and lower than 50 nanoscales, which is in line with TEM results (Figure 5). Moreover, the UV-vis spectrophotometer revealed that drug lodging and encapsulation efficiency of EDV were 37.45 ± 0.61% and 99.88 ± 0.01%, respectively.

### 3.4. Observation on Release Pattern of GSH-PMAA-EDV

**Figure 6:**
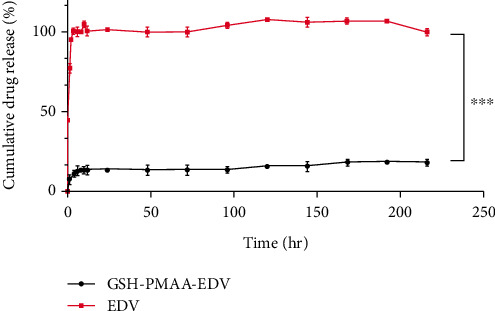
In vitro drug release profile of EDV and GSH-PMAA-EDV nanogel.

The release profile of the EDV drug from the fabricated nanogel as well as the free EDV is given in Figure 6. In this assay, in order to confirm that the dialysis bag release is not a rate-limiting factor in this method, the release of the pure EDV was also investigated. According to the release profile, EDV showed a burst release pattern in which about 98% of the EDV has been released from the dialysis bag into the external media, while in the first 10 hours, about 13% of the EDV is released from this nanosystem. After that, it reached the plateau area, and up to 216 hours, about 15% of the drug was released.

### 3.5. The Verification of Size and Zeta Potential of GSH-PMAA-EDV

**Figure 7:**
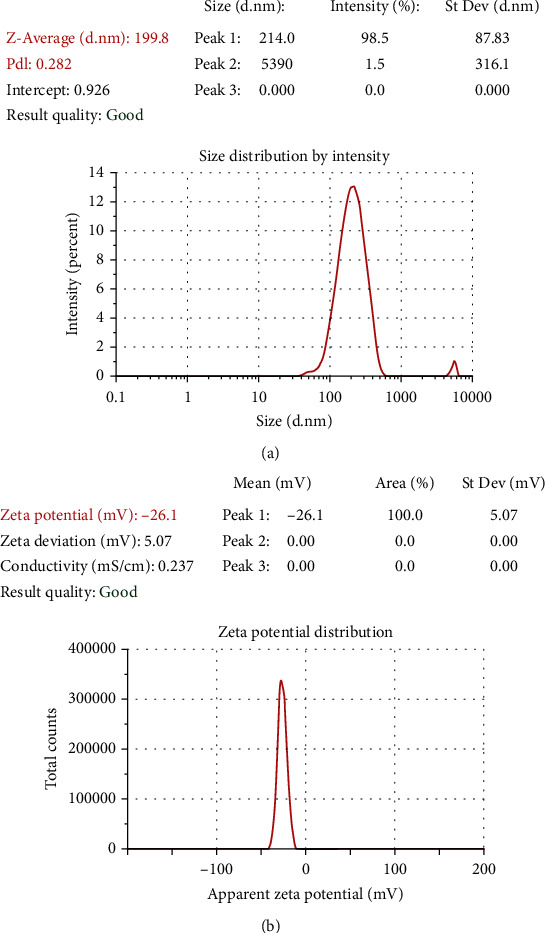
Size (a) and zeta potential (b) of GSH-PMAA nanogel.

As depicted in Figure 7(a), the mean hydrodynamic size and its corresponding polydispersity index (PDI) of GSH-PMAA-EDV nanogel were 199.80 nm and 0.282, respectively. As shown in Figure 7(b), the zeta potential of the final formulation was determined and found to be -26.10 mV.

### 3.6. Treatment with Nanosystem-Enhanced Cognitive Performance of Rodents

**Figure 8:**
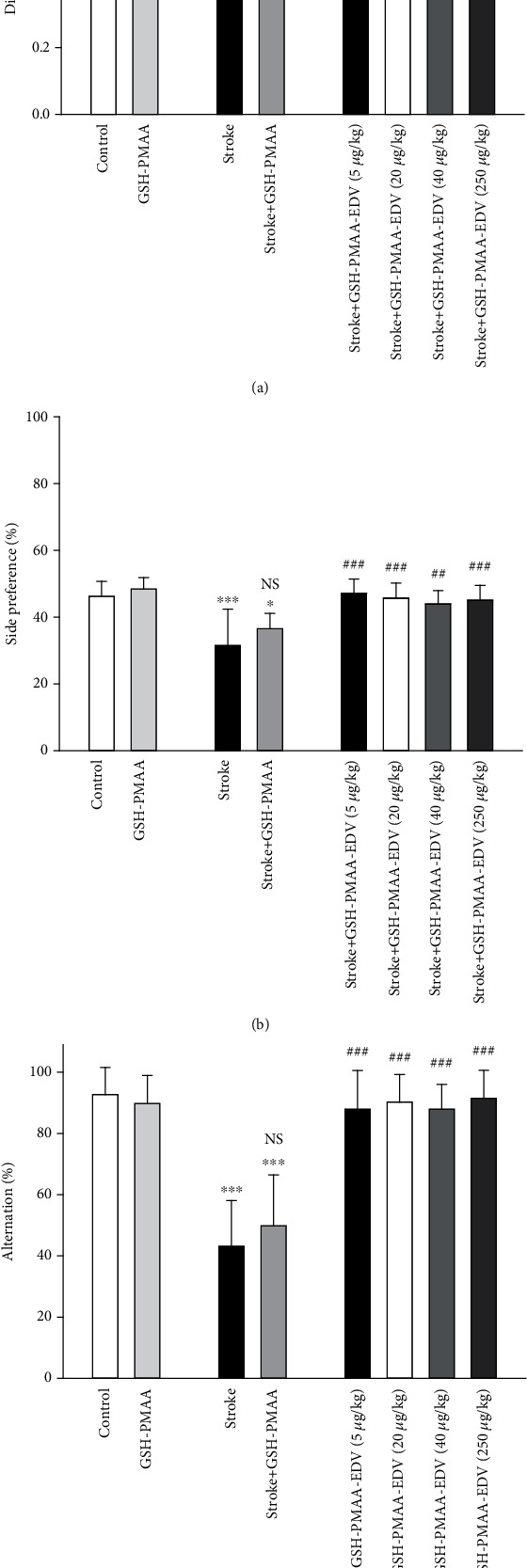
The effects of several doses of GSH-PMAA-EDV nanogel (5, 20, 40, and 250 *μ*g/kg) on behavioral function. (a) Discrimination ratio as an output of novel object recognition test. (b) Tendency of the rats to choose one arm in 6 trials in T-maze test. (c) Choosing correct arm by rat in T-maze test. Values are expressed as the mean ± SD and were analyzed using one-way ANOVA followed by Bonferroni's multiple comparison test (*n* = 7 − 8). ^∗^*P* < 0.05, ^∗∗^*P* < 0.01, and ^∗∗∗^*P* < 0.001 compared with the control group. ^#^*P* < 0.05, ^##^*P* < 0.01, and ^###^*P* < 0.001 compared with stroke rats (*n* = 7 − 8).

NOR test results are variable in terms of the type of animal and the protocol used. Herein, we adhered to nature protocol, choosing no habituation session and a 24-hour intrasession interval to assess memory traces in male rats. Time spent on exploring objects in familiarization test was measured for each animal, and the rats that could not reach the total time of 20 s exploration within 10 minutes were excluded from the analysis. As depicted in Figure 8(a), TGI induction has significantly decreased the discrimination index in the stroke group compared to control (*P* < 0.01). TGI-induced rodents, treated with GSH-PMAA-EDV, demonstrated significant enhancement in exploratory behavior of a randomly chosen novel objects, especially in 5, 20, 40, and 250 *μ*g/kg of GSH-PMAA-EDV (*P* < 0.01). The application of GSH-PMAA did not impose significant effect on the discrimination index in nonstroke rodents (*P* > 0.05).

### 3.7. Spontaneous Alternation and Preference in Enclosed T-Maze

Rats were released at the distal point of the start arm and were free to explore the maze and choose an arm (sample phase), then confined for 30 seconds, and extracted. After 2 minutes, rat was released at the same point and was free to choose one goal arm spontaneously for a maximum time of one minute. Otherwise (more than 3 minutes after choice phase), the rat was tested again after a few hours and on another day or excluded from analysis. As demonstrated in Figure 8(b), there is no significant difference with chance level (50%) which was observed in control and treated rat with only GSH-PMAA (*P* > 0.05). As demonstrated in Figure 8(c), stroke has caused a significant decrease in the number of correct alternations when compared to the control group (*P* < 0.001). Further application of GSH-PMAA-EDV in TGI-induced rats, namely, 5, 20, 40, and 250 *μ*g/kg, has managed to significantly improve the alternation factor (*P* < 0.01 or *P* < 0.001). The application of GSH-PMAA did not impose a significant impact on the alternation factor (*P* > 0.05).

### 3.8. GSH Level Alternation in Hippocampal Tissue

**Figure 9:**
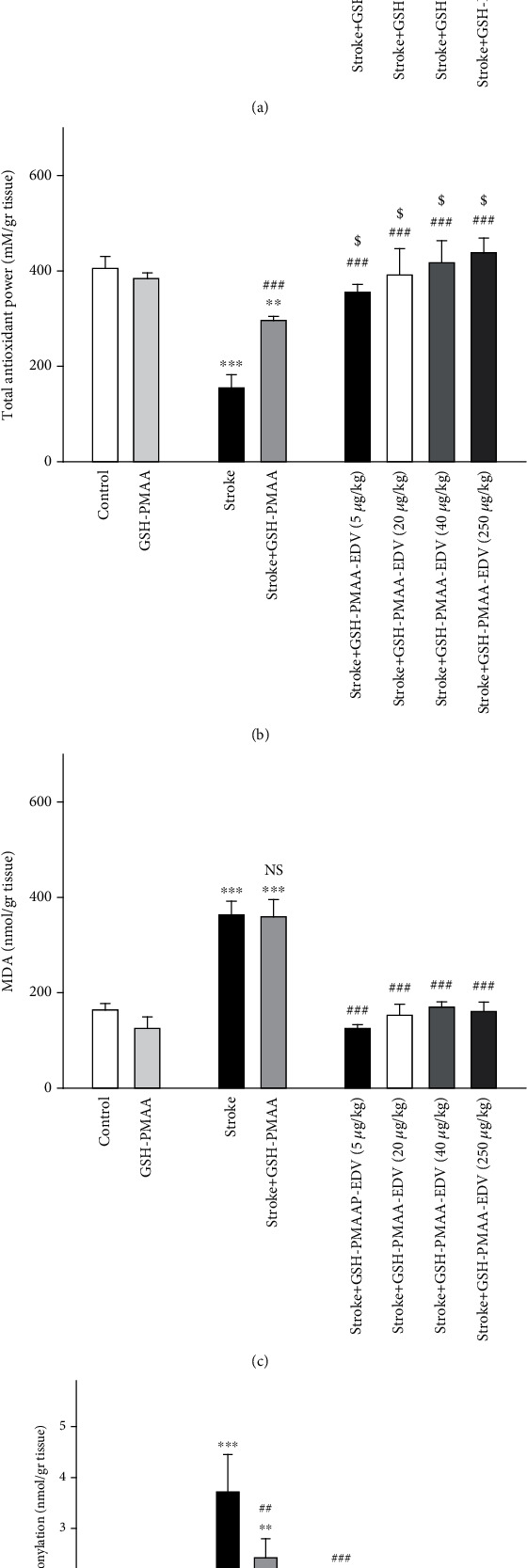
The effects of several doses of GSH-PMAA-EDV nanogel (5, 20, 40, and 250 *μ*g/kg) on biochemical alteration. (a) Reduced glutathione level, (b) FRAP level, (c) MDA level, and (d) protein carbonyl amount. Values are expressed as the mean ± SD and were analyzed using one-way ANOVA followed by Bonferroni's multiple comparison test. ^∗^*P* < 0.05, ^∗∗^*P* < 0.01, and ^∗∗∗^*P* < 0.001 compared with the control group. ^#^*P* < 0.05, ^##^*P* < 0.01, and ^###^*P* < 0.001 compared with stroke rats, and in case of GSH and FRAP levels, ^$^*P* < 0.05, ^$$^*P* < 0.01, and ^$$$^*P* < 0.001 were compared with the stroke-GSH-PMAA group (*n* = 3 − 4).

As displayed in Figure 9(a), stroke model in rodents has caused a significant drop in GSH levels when compared to the control group (*P* < 0.001). Significant improvement in GSH level was observed in the stroke group when treated with 20, 40, and 250 *μ*g/kg of GSH-PMAA-EDV (*P* < 0.001). While the stroke-GSH-PMAA has demonstrated no significant increase in GSH level compared to the stroke group (*P* > 0.05), all nano-EDV groups showed a significantly higher GSH level when compared to the stroke-GSH-PMAA group (*P* < 0.05). No significant alteration in GSH level was noticed when GSH-PMAA was applied to normal rodents compared to the control group (*P* > 0.05).

### 3.9. Imposed Effect on the Total Cellular Antioxidant Level

As illustrated in Figure 9(b), rodents under TGI method demonstrated a lower level of cellular antioxidants (*P* < 0.001). The aforementioned pattern has altered in stroke rats under GSH-PMAA-EDV treatment with all dosing groups (20-250 *μ*g/kg) showing significant improvement when compared to the stroke group (*P* < 0.001). The FRAP level has significantly increased in the stroke-GSH-PMAA group compared to the stroke group (*P* < 0.001), and this effect significantly amplified in treated group GSH-PMAA-EDV compared to the stroke-GSH-PMAA group. The FRAP value has not gone any change in the GSH-PMAA group when compared control groups, respectively (*P* > 0.05).

### 3.10. Significant Changes in MDA and PCO Levels

Figures 9(c) and 9(d) depict major rises in MDA and PCO levels which were noticed following TGI model of stroke compared to the control group (*P* < 0.001). The following treatment with 20-250 *μ*g/kg of GSH-PMAA-EDV has significantly ameliorated the surge of MDA and PCO levels when compared to the stroke group (*P* < 0.001). The application of GSH-PMAA in stroke-induced rodents has significantly decreased PCO level (*P* < 0.01); however, the same pattern was not observed in MDA factor (*P* > 0.05). The administration of 250 *μ*g/kg GSH-PMAA-EDV has not altered MDA and PCO levels when compared to the control group (*P* > 0.05).

### 3.11. Histopathological Assessment

**Figure 10:**
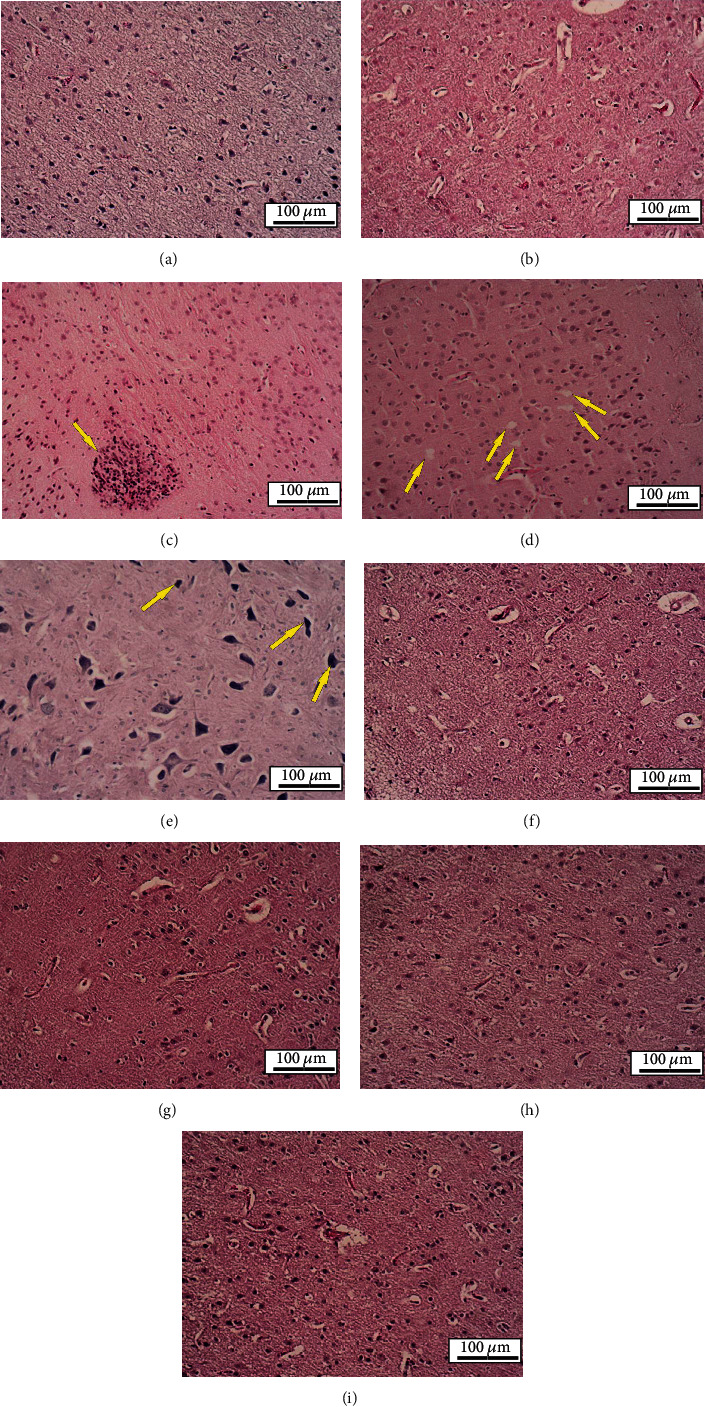
Histological sections of experimental groups' brain. H&E staining, ×100. (a, b) Normal saline and GSH-PMAA group, respectively: histological structure was normal. (c) GSH-PMAA-EDV 250 *μ*g/kg group: microglial nodule in the brain was observed (arrow). (d, e) TGI group: vacuolation and degeneration (arrows in (d)) and basophilic neuronal necrosis (arrows in (e)) were observed. (f–i) Received GSH-PMAA-EDV 5, 20, 40, and 250 *μ*g/kg groups, respectively: histological structure was normal.

As depicted in Figure 10, the vacuolation, degeneration, and basophilic neuronal necrosis have increased following TGI model for stroke. In contrast, the tissue damages decreased substantially after GSH-PMAA-EDV in all applied groups (5, 20, 40, and 250 *μ*g/kg). Microglial nodule in the brain was observed in animals receiving 250 *μ*g/kg of GSH-PMAA-EDV.

## 4. Discussion

The GSH-PMAA-EDV characterization has led to more sustained pattern for EDV release and higher penetration of this dosage form through the BBB. In addition, these effects were accompanied by demonstrating no toxicity in rodent at the highest applied therapy dose (250 *μ*g/kg). In addition, the loading of EDV into nanogel has managed to amplify the cellular antioxidant capacity and halted PCO surge compared to GSH-PMAA application on ischemic-like model.

Ischemic stroke is among one of the leading causes of mortality and malfunction among modern communities. The poststroke surge of oxidative stress and inflammatory cytokines are noticed to be the main factors in neural cell death [12]. The conventional and old-fashioned drug delivery approaches cannot be efficient in the treatment of various disorders such as stroke. This is due to the fact that probably drug molecules do not reach the desired site of action with an effective concentration or accumulate and distribute nonspecifically in other tissues or organs, and only a small part of the drug reaches the site of action, which may not be effective with this low dose and necessitates increasing the dose of the drug in order to achieve the therapeutic response. However, this approach increases drug toxicity and its unwanted side effects. In the case of EDV, the drug regimen requires an infusion for 14 days, and kidney impairment is a common side effect in adults with ischemic stroke adult cases being treated with 6 mg/kg EDV. In order to solve this problem, smart and targeted drug delivery was utilized, using a ligand that would deliver the cargo to the targeted tissue, which would increase concentration of the drug in intended site of action and enhance the therapeutic outcome [28]. TGI is one of the validated methods to investigate stroke-like behavior and biochemical response of rodents to pharmaceutical compounds [15]. Several free radical scavengers and natural antioxidants have been proven to be effective in neurodegenerative and neural damaging diseases [28–30]. GSH, a peptide composed of amino acids (cysteine, glycine, and glutamic acid), was utilized as a ligand for brain drug delivery which itself could slightly ameliorate oxidative stress in brain tissue.

In this study, GSH-PMAA nanogel was successfully fabricated and characterized by different characterization techniques such as FTIR, ^1^H NMR, TEM, and AFM. The carbodiimide method as a common condensing method was used to bind GSH targeting moiety onto the surface of PMAA nanoparticles through amide linkages. Activated carboxylic groups of PMAA were undergoing a nucleophilic attack of the amine groups present in the GSH molecules followed by amide formation. Bis AM is one of the main components in the preparation of gels and nanogels playing the role of a cross-linker, which is also widely used in industrial and chemical reactions [31]. We confirmed the formation of the conjugation between PMAA and GSH molecules using the FTIR characterization technique. These results along with ^1^H NMR spectrum of the GSH-PMAA showed all peaks related to both compounds (GSH and PMAA separately) in the final spectrum, which confirm that the polymerization reaction, nanogel formation, and targeting moiety conjugation have been successfully accomplished.

Moreover, we investigated the release pattern of EDV from the fabricated GSH-PMAA nanogel, which was in a controlled and sustainable manner. Therefore, this nanosystem can be used as a suitable drug carrier for the sustained delivery of class IV drugs such as EDV. It has been well documented that the size of the nanoplatforms not only affects the effectiveness of the fabricated drug delivery system but also is considered a major factor in determining its targeting ability, cellular uptake, and pharmacological effect [32]. As the size of the nanoparticle increases, its chance to cross the blood-brain barrier (BBB) decreases. In the other words, smaller particles have a higher chance to accumulate, penetrate, and internalize effectively into the brain compared to larger ones [33].

The zeta potential of drug vehicles is another fundamental parameter that directly measures the surface charge of a carrier, indicating the stability of colloidal nanosuspension. If all nanosuspension bear a negative or positive charge, the particles tend to repel each other than aggregate, and therefore, the stability of nanosuspension is improved. Considering the value of surface charge or zeta potential value representing the physical stability of colloidal systems, our nanosystem is highly stable. It is also worth mentioning that the surface charge of nanoparticles can highly affect the accumulation of developed nanogel in the bloodstream and even influence the interaction of cell layers or adhesion with developed nanogel. Interestingly, most of the blood cells and their components have a negative charge; therefore, the least interaction is expected for negatively charged nanoparticles with plasma. In other words, blood circulation time is improved in the case of negatively charged nanoparticles in comparison with the positively charged nanoparticles. Herein, we developed a negatively charged nanoparticle with high stability to avoid unnecessary interaction with biological components.

The application of GSH-PMAA-EDV has demonstrated significant improvement in rodents' discrimination and alternation factors in NOR and T-maze, respectively. While both aforementioned factors demonstrated a significant drop under TGI induction, the application of GSH-PMAA-EDV (20, 40, and 250 *μ*g/kg) has shown significant improvement in both behavioral tests compared to stroke-induced rodents, and no significant cognitive dysfunction was observed compared with the control group.

The number of correct choices in the T maze is under the direct impact of hippocampal damage [17]. Long-term potentiation (LTP) is mainly affected by hippocampal synaptic plasticity and facilitates synaptic formation [34]. The long-term trace memory was mainly affected by TGI, and the discrimination factor has improved significantly upon GSH-PMAA-EDV treatment since rodents recognized the former-encountered stimuli (object in the familiarization phase of NOR) which could be related to medial temporal lobe-ameliorated activity [35]. As for NOR, GSH-PMAA-EDV managed to improve animal working memory in T-maze, significantly.

In one related study, EDV has managed to suppress axonal injury in the corpus callosum, cortex, and CA3 zone of hippocampi, along with improvement in cognitive performance in the NOR test to the same level as the control group in terms of tendency to explore novel objects [36].

The TGI has caused a surge in free radical markers, namely, MDA and PCO, meaning the ischemia led to the energy production malfunction in mitochondria and triggered the accumulation of reactive oxygen species, which manifested itself by affecting unsaturated fatty acids and cellular proteins [16]. It seems that an increase in ROS levels subsequently leads to oxidative damage and pathogenesis of transient cerebral ischemia or stroke. The elevation in ROS production stimulated interaction with macromolecules and activation of cell death signaling pathways [21, 37].

EDV as a free radical scavenger has remarkable potency in the modulation of inflammatory factors such as tumor necrosis factor and interleukins [38, 39]. In this study, EDV-loaded GSH-PMAA has managed to attenuate the overproduction of MDA and PCO in all applied doses.

The overproduction of ROS also has led to the shortage of GSH and total cellular antioxidant levels, while an effective antioxidant defense is critical to prevent poststroke damage. GSH is in the forefront line of antioxidants, as mitochondrial GSH deactivates free radicals produced in the respiratory chain ether, playing a role as a cofactor or direct deactivator of ROS [40]. GSH-PMAA-EDV demonstrated recovering effect on GSH and FRAP values when applied above 20 *μ*g/kg and did not induce any toxic effect on GSH while GSH-PMAA-EDV was administrated 250 *μ*g/kg. Previous studies have demonstrated that increasing the GSH/GSSG ratio is associated with improving the superoxide dismutase activity in hippocampal tissue [41]. Finally, we aimed to evaluate the histopathological manifestations of the proposed nanoformulation to recheck the effectiveness and safety of GSH-PMAA-EDV in all applied doses (5-250 *μ*g/kg). GSH-PMAA-EDV has not induced any toxic histopathological adverse effect in CA1 site. In our previous study, mPEG-b-PLGA EDV nanoparticles also have not induced any toxicity in human neuroblastoma cell line while suppressing the expression of antiapoptotic genes [42]. The edaravone dosage used in this study was 100 times less than that of the calculated equal dose for rats, yet showing effectiveness and inducing no toxicity.

## 5. Conclusion

In conclusion, the fabricated GSH-PMAA-EDV nanogel offers more pronounced poststroke neuroprotection efficacy, providing more penetration to the BBB and more sustained release pattern while inducing no toxic effect in a rodent model in any of the administrated doses. The fabricated nanoparticle managed to suppress and modulate the surge of ROS byproducts and enhanced the level of intracellular antioxidants that is crucial in managing ischemic-like disorder.

### 5.1. Study Limitations

For further investigations, it is appropriate to focus on probable effect of GSH-PMAA-EDV on enzymatic antioxidant activity and the expression of neurotrophic factors and inflammatory genes like TNF and NLRP-2 to get a more generalized idea of how drug delivery could affect the function of edaravone in counteracting ischemic stroke in both biochemically and genetically impairment. The effect of GSH-PMAA-EDV on control rats could be investigated in further studies; the toxicology studies regarding to this nanoparticle and edaravone are critical in achieving more accurate perspective. Further, the result of the current work is limited to male rats which need for sure the beneficial effects of sex-specific nanogel EDV on both sexes in stroke condition.

## Data Availability

The data used to support the findings of this study are available from the corresponding author upon request.
